# Concerning the Bowel Affections of Infants

**Published:** 1876-01

**Authors:** Edw. Warren Sawyer

**Affiliations:** Lecturer on Obstetrics, Rush Medical College, Chicago


### CONCERNING THE BOWEL AFFECTIONS OF INFANTS.

*(Suggested by Prof. N. S. Davis's paper.)*

By EDW. WARREN SAWYER, M.D.,

Lecturer on Obstetrics, Rush Medical College, Chicago.

(Read before the Chicago Society of Physicians and Surgeons, Sept. 13, 1875.)

Mr. President and Gentlemen:

I desire at this time to occupy a moment with some consideration of the causation of the intestinal affections of infants, elicited by Prof. Davis's paper read before this Society at its last meeting.

And first, in reviewing the various bowel troubles of children, I believe the gravest error will be committed if these several diseases are considered collectively, either in their etiological or pathological relations, as though they possessed the same pathology and have the same causation. I hope to remind you that the intestinal affections of young children have marked individuality, and that a comprehensive knowledge of their characteristics only will enable us to treat these diseases rationally.

There is another chance of serious error in founding theories upon the showing of the mortuary tables of a city ; this source of error is so apparent as to seriously invalidate the conclusions drawn from these tables. Assuredly, the mortality tables may show, in an indefinite and general manner, that the bowel diseases of infants are more common in the summer than during the winter ; but the exacting student will not be satisfied with information so vague, concerning this large class of diseases : he wishes to know if a particular, well-marked disease is more prevalent during a particular state of the weather, in order that, by consulting the meteorological or other records for that period, he may learn whether or not the existing atmospheric and telluric conditions can stand in the relation to that particular disease as cause to effect. It is in this direction alone, that trustworthy information can be obtained ; but the present mortality records are utterly valueless for this purpose, because the giving of certificates of the cause of death is not exclusively in the hands of competent, honest practitioners.

I propose to examine briefly the more common of these affections, to see if they do really possess certain etiological distinctive traits ; and, for simplicity, I will divide them into two classes. In the first class, cholera infantum and dysentery will stand alone. For the present, one can not question the propriety of classing the cholera of infants among bowel complaints, and it may, in reality, have as much claim to be so classed as typhoid fever has to its place among enteric diseases ; but subsequent study may remove both from this category, and call them specific toxæmiæ.

An infant living in the poor and badly drained wards, or the more healthy localities, previously of robust health, or a puny child, is attacked suddenly with vomiting and discharges from its bowels, accompanied by abdominal pain, a colic, and cramps of the lower extremities. The matter at first vomited is the food incompletely digested; if vomiting continues, greenish mucus is ejected with serous matter. The first dejections are the contents of the intestine; then follow in rapid succession large discharges of serum, and fœtid, watery matter containing small flakes and cells of epithelium from the lining of the intestine—"the rice-water dejections." If the onset is severe, the stage of collapse soon follows; the nose is pinched, the cheeks fall in, and the eyes are sunken; the lips are purple, and dark blood remains under the finger nails; the body is limp, and the extremities fall helplessly if raised; the expirations feel cold to the hand; the cutaneous surface is cool and clammy. From this moment, one of three courses is consecutive: first, cessation of the vomiting and intestinal transudation, recovery from the collapse, and convalescence within twenty-four hours; this course is the rarest. Second, death from collapse within forty-eight hours of the onset; next in frequency. Third, recovery from the collapse, follows, but loose dejections continue, and a diarrhœa is kept up by the presence of imperfectly digested food. Serious organic change has taken place in the intestinal lining, which favors the diarrhœa, and from which recovery is slow. In a day or two, the dejections contain disintegrated coagula of caseine from the milk, made green with changed bile, and mixed with some intestinal mucus; constituting the "chopped grass" dejections of some observers. I am persuaded this is the most common ending of cholera infantum, viz., in a diarrhœa which may soon end in death, or recovery after a long convalescence.

It is important to observe that the duration of cholera is short, it is usually limited to forty-eight hours, and ends with the collapse; the diarrhœa which is often a sequence, is from chronic indigestion, and is not essential to the first attack.

All have observed the identity of this disease with the cholera nostras, or morbus of adults; as far as we know, it is the same both respecting its causation and pathology. But, just as typhoid fever may occur without epistaxis and rose spots, even, so may the cholera of infants be modified and its vomiting be absent; on the other hand, just as there can be no typhoid fever without involving the patches of Peyer, so there is no cholera infantum without the sudden transudation of the serum of the blood through the intestine. This is its characteristic, but whether its essential pathology lies in the presence of bacteria or a blood ferment, we can not perhaps declare to-day.

I insist upon the identity of this disease because it has an identity, which it is both practicable and easy to recognize. One fact must have been suggested to you, viz., that the disease is not a common one; still, in the aggregate, many children are destroyed annually by cholera, and that, too, in the country and the populous cities.

Respecting the real causation of cholera infantum, we must confess our utter ignorance, for nothing is positively known of it. If it be accepted that cholera is a specific disease, (and who will say it is not?), then a specific origin must be admitted, for the day has long passed when several distinct origins were admitted for a single specific disease. A notable illustration of this is seen in the recent progress made in the study of typhoid fever, to which I have already alluded. It was formerly thought that filth, and the presence of decomposing animal and vegetable matter, were all that was required to engender this disease, but it is now known that one may live in a village of slaughter-houses, surrounded by foul privies and stinking sties, and yet not have typhoid fever. But once spread the dejections from a typhoid fever patient in that locality, and an epidemic of that disease will be precipitated which will not be limited to the unfortunate inhabitants of that unwholesome region. The development of the cholera of infants and adults, is obedient to the same law of extension, and the germs of cholera, if implanted in the proper soil, will bring forth cholera as immutably as the grain of corn brings forth corn and no other grain.

Many observers, with Prof. Davis, have noticed a greater prevalence of cholera infantum during the heated terms, than during the remainder of the year ; *ergo*, the heated atmosphere is the origin of cholera infantum. This conclusion is as fully shown to be erroneous, as the filth origin of abdominal typhus ; by the fact that cholera is not most common in the hottest climates, and that it not infrequently occurs in our own country at another period than the hottest. The first case of cholera infantum, which I saw with my teacher, occurred early in May, before one hot day had elapsed. As a matter of fact, quite as many observers have remarked the greatest extension of cholera, not during the hottest weather, but when the hot days are interrupted by cool nights, and by an occasional cold, wet day.

Further, we are told that if the degree of heat is not the direct source of cholera, it is something else which is aroused, set in motion by the caloric. One can not oppose this theory with much that is positively known, but there is as much reason for supposing that the dew, the moonshine, the wind, or any other imponderable agent, may be the source of this disease.

In view of the facts, it is clear that the occurrence or extension of cholera infantum, does seem to be favored by some condition connected with the warm semester of the year ; perhaps in the same manner as that in which small-pox is favored by winter ; but the many instances of the occurrence of these diseases at other seasons, show conclusively that their occurrence or propagation does not depend upon any degree of temperature.

Closely allied to cholera infantum, is the so-called choleriform diarrhœa, to which many infants are subject during the prevalence of cholera, and which attacks adults, even, while sporadic or epidemic cholera is prevalent. In this affection, the child has been subjected to, and infected by the poison, but is overcome by it to a less degree than one who is the victim of well developed cholera. Nor does this imply an untenable theory, but a feature in infectious diseases that every one recognizes. The disparity in the effect of infectious poisons is explained in two ways: first, that it depends upon the amount of poison taken into the circulation; second, upon the vulnerability of the person. I accept the latter, for it agrees more closely with our knowledge of other intoxications.

The dysentery of infants I have also included in the first class. As the object of these remarks is to define and separate the intestinal disorders, but little need be said of dysentery, for all have admitted for it a specific causation; and that it is not essentially unlike the dysentery of adults. Being an infectious disease which occurs usually in endemics or epidemics, it will not be entertained that any degree of heat can cause dysentery; it is sometimes entirely absent throughout the hottest summers; when it occurs, however, it has sometimes been observed to select the summer months; though its period of most rapid extension is after the occurrence of frosts, and the beginning of cool nights.

Reliable data are wanting concerning the relative prevalence of dysentery; most observers agree in this, however, that it is not a common disease of infants.

These remarks are not to be considered as applicable to entero-colitis, a disease whose morbid anatomy is essentially that of dysentery, but whose etiology differs from that of the latter in this most important particular, viz., entero-colitis is not a specific disease; hence, strictly, it should be included in the second category.

A variety of exciting causes are sufficient to set up an inflammation of the colon with the lower part of the small intestines, but the most frequent cause is the retention of irritating and undigested matters in the colon; and because the habit of constipation is necessary to the habitual retention of fæces in the colon, it follows that those persons who labor under the habit of constipation are more liable than others to this disease. Further, because constipation among infants is rather the exception than the rule, they are not the especial subjects of entero-colitis, as nearly all will admit when its characteristics are recalled: tormina and tenesmus are prominently present from the first; in a day or two, the tenesmus is usually so constant that almost the entire day is spent in straining at stool; the lower sphincters of the bowel soon become paralyzed, the anus excoriated, open and everted. The character of the dejections is of the greatest diagnostic importance; when the bowels have been once completely evacuated, the dejections contain blood and pus, and soon but little else; this is the essential factor; which, if absent, excludes the disease.

I have noticed one striking fact in consulting authors, viz., that no one has accorded any importance to the agency of heat as a causation of entero-colitis; on the contrary, almost every writer says that exposure to cold and damp is an exciting cause of this disease.

In the second class of bowel disorders, I would include those of simple diarrhœa. At the outset, we must admit that it is not scientific to group together a class of diseases whose causation is obviously so dissimilar, and which have nothing in common except the diarrhœa; but, in the present state of our knowledge, we can perhaps adopt no better classification, except with the malarial diarrhœa, which might be included with other diseases resulting from malarial toxæmia; while the reflex diarrhœa of dentition might, with some propriety, be classed with the neuroses.

The first of the bowel affections I would review, in which diarrhœa is the prominent symptom, is the reflex diarrhœa of dentition. Reflex phenomena are everywhere common enough in medical experience: thus the presence of undigested matter in the stomach and bowels has not infrequently excited convulsions in an infant, and has been followed by paralysis of a limb. A recent medical journal contains an instance of amaurosis in a child from the presence of a tape worm. These are phenomena due to reflection outward from the bowel, but instances of the opposite reflection are not wanting. I know a lady who has a watery diarrhœa, sometimes amounting to eight dejections in an hour, each time a thunder shower passes over her head. I have seen more than one man whose intense anxiety on the eve of a battle, gave him a violent diarrhœa; under the same circumstances, instead of the rapid intestinal transudation, other men pass large quantities of urine. These are instances of reflection from profound moral impressions, but others are known quite similar to the disease under consideration. Thus it is not uncommon for an adult or child to have a diarrhœa, caused directly by the pain of a felon; or a little child suffers from a finger bruised in the jamb of a door, and has a watery diarrhœa as a consequence.

Two essential conditions seem necessary for the occurrence of this affection: first, the vulnerable subject; second, a profound impression upon, or irritation of some part or region of the body which is well supplied with nervous structures. How fully these conditions are answered in the teething child must be apparent to all.

Besides the profound nervous impression that the growth and eruption of the teeth is capable of exciting, it is important to recall the fact that, simultaneously with the process of dentition, another physiological change is taking place in the intestinal structure itself, viz., a development, an evolution of the follicular apparatus of the intestine, which, in itself, may excite a diarrhœa by inducing an hyperæmia of the intestinal lining.

The great majority of writers have fully admitted this variety of infantile diarrhœa, and I would hardly be warranted in thus enlarging upon it, had not the author to whom I refer, entirely ignored and repudiated this affection in the paper which has been presented to you. He says, children teeth in the winter as well as in the summer, and that teething cannot cause cholera infantum, because the winter mortuary tables show almost no cholera infantum, while hundreds of children die from this disease during the summer. I am not aware that any one has affirmed that the process of teething is the cause of cholera infantum, but a reflex diarrhœa, which is essentially unlike cholera. Let me remind you, also, that the tables furnished by the Board of Health do not aim at showing the prevalence of a disease, but only the deaths from a disease; and that in an unreliable manner, as we have seen, because of the unreliable data furnished to the Board by promiscuous practitioners. Now as the diarrhœa of teething is early amenable to proper treatment, and it is hoped that but few die from this diarrhœa alone, it is not to be expected that the mortuary tables can inform us of the relative prevalence of this disease at different seasons of the year.

The accuracy of another premise might be questioned by some, viz., that as many children are teething in the winter as in the summer. It has been found that a larger number of births occur during the last two and first two months of the year than at other seasons. If this be true, then the majority of infants are cutting their first teeth nearly simultaneously during the summer season. This is comparatively unimportant, while the great fact remains established, that the process of dentition does provoke diarrhœa in infants, and that, too, in the winter as well as in the summer, a fact which all can verify. But as all are not susceptible to reflex phenomena, so do some children pass through dentition at all seasons without diarrhœa. Moreover, many infants suffer this affection who are not seen by the physician; the mother, recognizing the trouble, says "the baby is teething," and contents herself with rubbing the swollen gum till the tooth comes through, and there is a slight respite until the gum becomes tender again from another offending tooth.

Again, the essayist declared the process of dentition to be like the growth of any bone. I beg that gentleman to pardon me, but the notion seems as preposterous as it is incorrect. What analogy can he recognize between the unfelt and unnoticed consolidation of the epiphyses and shafts at fifteen, and the sickening, weakening process by which the wisdom tooth is erupted at twenty! No such analogy exists in the adult, and still less in the sensitive infant, who is overcome by the painful yet physiological process of dentition.

It is highly probable that the heat of summer, by its relaxing, enervating influences, may favor the continuance of all varieties of diarrhœa; but it must be accepted by you that the disease under consideration is unequivocally the reflex effect of the process of dentition, and can not be set in motion by heat.

Second: The diarrhœa of sepsis or toxæmia. I have not wished to coin a new term for a disease with which you are all familiar; but, following the plan already expressed, we will ascribe to each disease its own characteristics, so far as we are able to do this accurately as in the present instance.

The continued respiration of air which is vitiated by the exhalations of decomposing organic matters, will produce in some persons a toxæmia, that declares itself, among other ways, in a diarrhœa. A common illustration of this, is the diarrhœa which some medical students suffer who imprudently remain too continuously in the dissecting and autopsy room. An interesting fact in this connection is, that the dejections exhale a foul effluvium quite suggestive of that from the cadaver.

Among infants, the usual source of this toxæmia is in respiring the air loaded with exhalations from privies, from the decaying matter left upon the surface, and from stagnant pools of water made foul by receiving slops and offal of all kinds. It follows from this that infants living in poor wards, and in districts whose sewerage is incomplete, should e the special subjects of this disease; moreover, it is this disease, the prevalence of which seems to stand in a direct ratio with the amount of pavement, or the extent of drainage of a certain precinct—it is this disease, and not cholera usually, that increases the infant mortality of large cities and towns especially.

As the heat of summer favors decay and the dispersion of noxious matters, it is natural that the period of greatest prevalence should be coincident with hot weather. But heat obviously can not be the primary origin of this affection, for let the children be completely removed from this vitiated atmosphere, and this disease will not occur, no matter how hot the summer. It is also well known that if the drain pipes of a house, in a most favorable locality, become obstructed, the infants in that house stand in imminent danger of this disease even in mid-winter.

Third: The diarrhœa from acute indigestion. The infant is profoundly and easily overcome by the effects of matter taken into the stomach, which is itself indigestible or capable of intercepting the process of digestion. In the case of the child whose food is mixed, and who eats fruit and vegetables, ripe and unripe, and improperly cooked meats, the exciting causes of the attack of indigestion are as various as in the adult; but in the infant whose only food is milk, either from the mother or cow, we are forced to look to the milk as containing the offending matter.

An interesting physiological inquiry is raised at once: Can the milk of a healthy cow contain matters almost toxic in their effects upon the infant whose stomach receives it? Most practical men will answer this affirmatively, almost without thought. As a familiar fact, all know that a cow fed on garlic will give milk flavored with garlic. If we were acquainted with no other proofs, this alone shows conclusively that certain matters, taken into the stomach of an animal, are eliminated through her milk ducts; if this is true of garlic, why not of other matters that we are not able to detect. In support of this, I recall one instance, which can be supplemented with many others by nearly all present. An infant less than six months old had been fed exclusively upon the milk of one cow, diluted as is recommended, and had suffered a diarrhœa from the first, which had resisted all remedial measures. I inspected the surroundings of the cow stable, and found that, it being mid-winter, the cow had been fed largely upon old, wilted cabbage leaves; the milk from this source was immediately suspended, and the recovery of the infant rapidly followed. Two months later, an accident happened to the vessel holding the daily allowance of the child, and the little one was fed upon the milk from the first cow, for a half day only, but that was sufficient to provoke a diarrhœa the following night, which continued some days.

In the pastures, there are many sources of contamination of the cow's milk; she drinks from little stagnant pools of water upon the surface, that are the receptacles of much decomposing matter and offal, washed in by every shower. You will observe that the time of the greatest danger of contamination of the water in the pastures, and the cow's milk, is during the heated term, when the noxious matters are concentrated by the rapid evaporation of the water. Hence the prevalence of diarrhœa from this cause during the summer, though it frequently occurs during the winter, as in the case cited.

A notable instance of the effects of contaminated drinking water occurred in this city very early during the past spring. Before the breaking up of the ice on the lake, it was said that the overflowing river had cut a channel through the ice in such a direction that the foul river water was driven toward the crib, and, without doubt, became mixed with the water in the city mains. However, in the space of twenty-four hours, I saw three cases of diarrhœa, accompanied by an unusual amount of depression, when I had not seen a case of diarrhœa for weeks previously. Upon inquiry of my colleagues,

I learned that a sudden outbreak of diarrhœa had occurred in their practice also. If there was no apparent increase in infantile diarrhœa at that time, the fact may be explained by remembering that young infants drink but little water ; and that the water perhaps was too slightly contaminated to affect the milk of the mother or cow who drank it.

It is not improbable that the infant who is fed naturally upon the breast, may also have a diarrhœa from vitiated milk. If cabbage leaves eaten by a cow can impart an injurious ingredient to her milk, why may not the mother's milk be thus changed ?

Fourth : In a few instances, a form of gastro-intestinal irritation among children has fallen under my observation, with which I was practically unacquainted. The slightly marked periodicity of the affection suggested the giving of quinine, which I did with benefit. I have since concluded that there is among infants, as I know there is among adults, a diarrhœa dependent among malarial infection, in which an anti-periodic, and especially quinine, seems indicated.

Fifth : I should not omit the familiar form of intestinal catarrh, which is the immediate effect of exposure to cold and damp, and which occurs more frequently during the summer and fall ; because the exposure is more common during those seasons.

The bowel affection, which is, by far, the most frequently presented to the physician, is diarrhœa from chronic indigestion. This is not a disease, *per se*, but the result of many affections. Thus it may be the sequence of cholera or entero-colitis, or the continuance of the reflex diarrhœa of teething, or an attack of acute indigestion may have been its beginning. I had almost said it is not important what the disease was that gave origin to this affection, the pathology and indications are essentially the same. It is now a diarrhœa kept up by the imperfect performance of digestion. Some disease, usually one of the foregoing, has effected some serious organic change in the bowel, or an atony which does not admit of perfect digestion. The continuance of this diarrhœa seems favored by the depressing influence of hot weather ; during which the little patient becomes rapidly emaciated, and is soon destroyed.

Conclusions : Upon examination separately of the diseases known as bowel complaints, we have failed in every instance to find that the disease was originated by heat ; therefore heat can not be regarded as the source of the several diarrhœas ; for what is untrue of each of a class is untrue of the whole.

It is unscientific to apply the results of study of one particular affection to the entire class of intestinal diseases, and it is the ignoring of this source of error which has led to such varied and wrong conclusions. Thus, for example, many have noticed that bowel affections are more prevalent in summer, and have concluded that the origin of these diseases is in the heated atmosphere. Another observer, whose field is the poor and foul wards of a city, concludes that the bowel affections of children are caused by foul air. A third has the fortune to see much diarrhœa among teething children, and hence concludes that dentition is the cause of all the bowel affections of infants. A fourth has observed that those children who nurse from a bottle, are often the subjects of diarrhœa, and concludes that artificial feeding is the cause of the diarrhœa of infants. In point of fact, these different observers have been studying distinct diseases.

Finally, the comprehension and rational management of this important class of disorders can only be attained when we recall the fact, that the causes of infantile diarrhœa are as distinct and numerous as these different affections themselves.

